# Endoplasmic reticulum stress preconditioning modifies intracellular mercury content by upregulating membrane transporters

**DOI:** 10.1038/s41598-017-09435-3

**Published:** 2017-09-28

**Authors:** Fusako Usuki, Masatake Fujimura, Akio Yamashita

**Affiliations:** 1 Department of Clinical Medicine, National Institute for Minamata Disease, Kumamoto, 867-0008 Japan; 2 Department of Basic Medical Sciences, National Institute for Minamata Disease, Kumamoto, 867-0008 Japan; 3 Department of Molecular Biology, Yokohama City University School of Medicine, Yokohama, 236-0004 Japan

## Abstract

Endoplasmic reticulum (ER) stress preconditioning protects cells against methylmercury (MeHg) cytotoxicity by inducing integrated stress responses such as eIF2α phosphorylation, ATF4 accumulation, and nonsense-mediated mRNA decay (NMD) suppression. Here we demonstrated that ER stress preconditioning results in the upregulation of membrane transporters, leading to a decrease in intracellular mercury content. Our analyses showed that ER stress preconditioning upregulated the expression of methionine transporters that affect the cellular influx of MeHg, LAT1, LAT3, and SNAT2; and a membrane transporter that affects the efflux of MeHg, ABCC4, in MeHg-susceptible myogenic cells. Among these, ABCC4 transporter expression exhibited the greatest elevation. The functional significance of ABCC4 transporter in the efflux of MeHg was shown by the ABCC4 inhibition study. Additionally, we identified the role of phospho-eIF2α/ATF4 pathway in the upregulation of LAT1, SNAT2, and ABCC4 and the role of NMD suppression in LAT3 upregulation. Further, we detected that ER stress preconditioning amplified membrane transporter expression most likely through the translation of the upregulated mRNAs caused by ATF4-dependent transcription and NMD suppression. Taken together, these results suggested that the phospho-eIF2α/ATF4 pathway activation and NMD suppression may represent therapeutic targets for the alleviation of MeHg cytotoxicity by enhancing mercury efflux besides inducing protective stress responses.

## Introduction

Methylmercury (MeHg) toxicity is a continuous environmental risk to human health. The critical role of oxidative stress in the pathogenesis of MeHg toxicity has been clarified both *in vitro* ^1–6^ and *in vivo* ^7–9^. Failure to protect cells against the early oxidative stress triggers subsequent endoplasmic reticulum (ER) stress and apoptosis^5^, suggesting that protective factors against oxidative and ER stress may be important in combating MeHg cytotoxicity. We previously demonstrated that the disturbance of cellular redox systems and the incidence of oxidative stress are caused by posttranscriptional defects of the major antioxidant selenoenzymes under MeHg-induced relative selenium-deficient conditions, and that treatment with ebselen, a seleno-organic compound, effectively suppressed oxidative stress and protected cells against MeHg-induced cytotoxicity^10^. We further demonstrated that ER stress preconditioning also protects cells against MeHg toxicity through the induction of integrated stress responses (ISR) including phosphorylation of eukaryotic initiation factor 2 alpha (eIF2α), accumulation of activating transcription factor 4 (ATF4), and suppression of nonsense-mediated mRNA decay (NMD)^11^. Although our previous study showed that ATF4 accumulation and ISR-induced upregulation of glucose regulated protein of 78 kDa (GRP78) under ER stress preconditioning are critical for the protection against MeHg-induced cell damage, the specific mechanism by which ER stress preconditioning-induced ISR protects cells against MeHg cytotoxicity remains to be fully elucidated.

It has been reported that ISR regulates the expression of genes involved in amino acid import^12^. Notably, MeHg is readily incorporated into the sulfur-containing amino acid cysteine to form a MeHg-cysteine complex because MeHg has a high affinity for the sulfur atom in thiol groups. As the MeHg-cysteine complex conformationally mimics the essential amino acid methionine, the complex can be transported to the cells via amino acid transporters for methionine^13–15^. Therefore, ER stress preconditioning may affect intracellular Hg concentration through changes in the gene expression of methionine transporters. Additionally, the ATP-binding cassette (ABC) transporter cassette C subfamily 4 (ABCC4) protein, which functions in the efflux of glutathione conjugates, has been shown to be associated with the cellular efflux of MeHg as well^16,17^.

In turn, NMD represents an mRNA quality control mechanism that detects the premature termination codon (PTC) located 5′-upstream of the last exon–exon junction and degrades PTC-containing mRNAs^18^. NMD suppression study suggested that NMD regulates transcripts with amino acid metabolism and transport functions that include upstream open reading frame(s) (uORF), alternative splicing that introduces nonsense codons, or frame-shifts and introns in the 3′untranslated region^19^. Furthermore, *Abcc4* has been reported to have alternatively spliced transcripts (*Abcc4s*) bearing nonsense codons. Together, these findings suggest that NMD suppression caused by ER stress preconditioning may lead to the change in such membrane transporters mRNA expression. Our previous study showed that NMD suppression upregulates ATF4 mRNA, suggesting that ER stress preconditioning amplifies the accumulation of ATF4, a key transcriptional activator involved in adaptation to stresses, through NMD suppression-mediated increase in ATF4 mRNA and eIF2α phosphorylation-mediated translation facilitation of ATF4 protein-coding ORF^11^. Therefore mild ER stress-induced ISR may play a role in changes in the gene expression of membrane transporters.

Here we investigated the effect of ER stress preconditioning on the expression of these membrane transporters and on intracellular Hg concentration following exposure to MeHg using MeHg-susceptible myogenic cells. The role of mild ER stress-induced ISR in modifying the expression of these membrane transporters was also determined.

## Results

### Effect of TPG pretreatment on intracellular Hg concentration and the expression of membrane transporters following MeHg exposure

**Figure 1:**
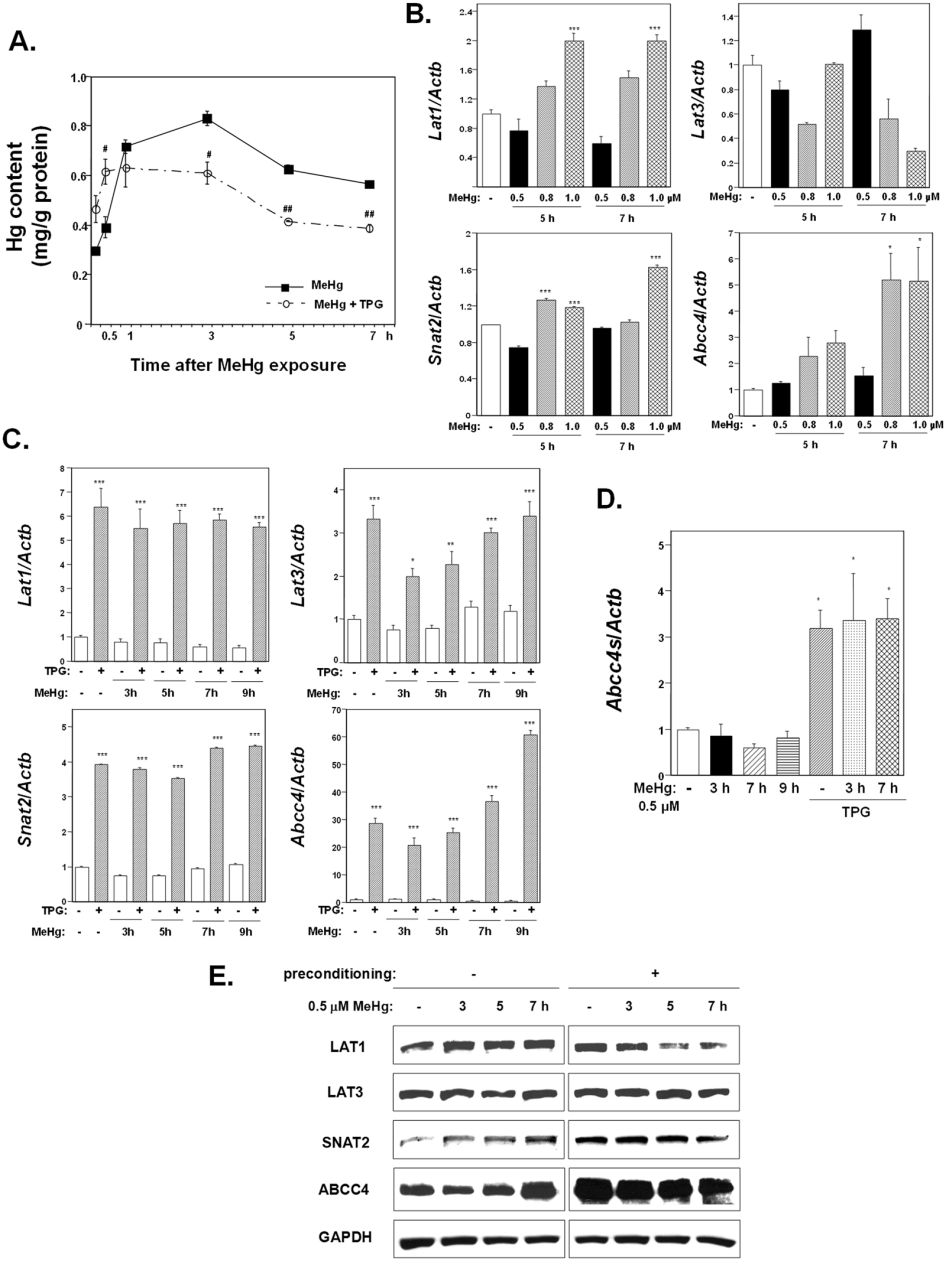
Effect of ER stress preconditioning on intracellular Hg content and on the expression of membrane transporters following exposure to MeHg. (**A**) Time course study of intracellular Hg content. C2C12-DMPK160 cells pretreated with TPG (0.3 μg/ml) for 16 h were exposed to 0.5 μM MeHg. Values represent the means ± SE (n = 3). ^#,^^##^Significantly different from TPG-untreated cells by a one-way Welch’s *t*-test (^#^p < 0.05, ^##^p < 0.01). (**B**) Changes in the expression of mRNA following exposure to MeHg. Total RNA was extracted from cells treated with 0.5, 0.8, or 1.0 μM MeHg for 5 or 7 h and membrane transporter mRNA expression was analyzed by RT-qPCR. The histogram depicts the indicated mRNA normalized to *Actb* mRNA. Values shown are the means ± SE of 3 separate experiments. *^,^ ***Significantly different from MeHg-untreated cells by a one-way ANOVA (*p < 0.05, ***p < 0.001). (**C**,**D**) Expression of membrane transporter (**C**) or the alternative ABCC4 splice-form (*Abcc4s*) (**D**) mRNA was analyzed at the times indicated after the exposure to 0.5 μM MeHg using RT-qPCR. The histogram depicts the indicated mRNA normalized to *Actb* mRNA. Values shown are the means ± SE of 3 separate experiments. *^,^ **^,^ ***Significantly different from TPG- and MeHg-untreated cells by a one-way ANOVA (*p < 0.05, **p < 0.01, ***p < 0.001). (**E**) Western blotting analyses of membrane transporter expression. Total cell lysates prepared at the times indicated were analyzed using the indicated antibody probes. Cropped blots are shown; all gels were run under the same experimental conditions.

Intracellular Hg concentration increased soon after the exposure to MeHg in mouse myogenic MeHg-susceptible C2C12-DMPK160 cells. The influx of MeHg was more rapid in cells pretreated with 0.3 μg/ml TPG for 16 h than that in non-preconditioned cells (Fig. 1A). The peak of Hg concentration occurred 3 h following MeHg exposure in non-preconditioned cells but at 1 h post-exposure in preconditioned cells. Furthermore, the efflux of MeHg was also more rapid in cells pretreated with TPG than in TPG-untreated cells. These data suggested that the efflux of MeHg was more highly activated than the influx following MeHg exposure and that ER stress preconditioning further enhanced this phenomenon thus alleviating the load of MeHg on cells.

Next we examined the mRNA expression of methionine transporters related to the influx of MeHg, L-type amino acid transporter (LAT) 1, LAT3, and sodium coupled amino acid transporter 2 (SNAT2)^14,20–22^; and ABCC4 related to the efflux of MeHg. As shown in Fig. 1B, quantitative reverse transcription polymerase chain reaction (RT-qPCR) analyses demonstrated that the exposure to MeHg caused an upregulation of the mRNA expression of *Lat1*, *Snat2*, and *Abcc4* in a dose-dependent manner, but not of *Lat3*. Among these, *Abcc4* upregulation was markedly higher. ER stress preconditioning further upregulated the gene expression of all of these membrane transporters with *Abcc4* mRNA levels being exceptionally high (Fig. 1C). An alternative splicing variant of *ABCC4* (*ABCC4s*) bearing nonsense codons was also upregulated (Fig. 1D). The results of western blot analysis showed an increase in the expression of LAT1, SNAT2, and ABCC4 in preconditioned cells compared to non-preconditioned cells. Treatment with MeHg modified the expression of the membrane transporters, and especially enhanced ABCC4 proteins in non-preconditioned cells (Fig. 1E). Notably, the difference in intracellular Hg content between preconditioned and non-preconditioned cells corresponded to the changes in the expression of these transporters (Fig. 1A,C,E).

### mTOR expression in cells exposed to MeHg

**Figure 2:**
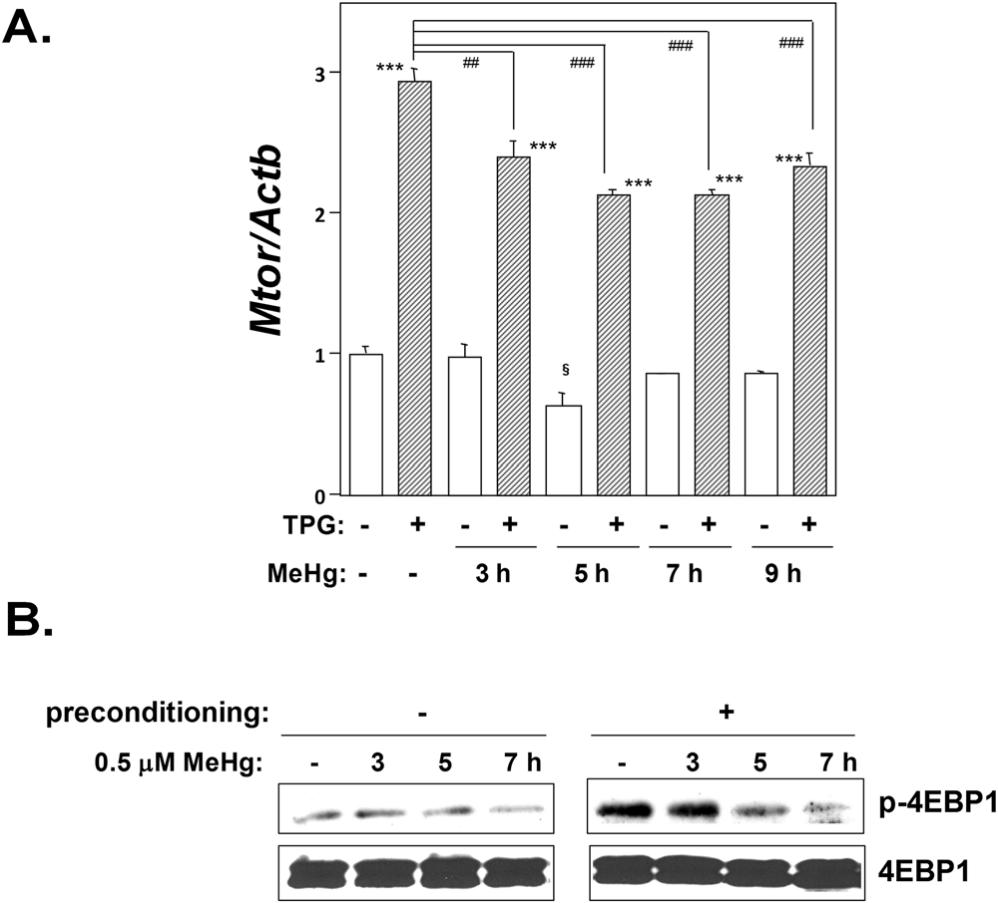
Effect of MeHg exposure and ER stress preconditioning on mTORC1 signaling. (**A**) Effect of ER stress preconditioning on mTORC1 signaling following exposure to 0.5 μM MeHg. The histogram depicts *Mtor* mRNA normalized to *Actb* mRNA analyzed by RT-qPCR. Values shown are the means ± SE of 4 separate experiments. ***Significantly different from TPG-untreated cells by a one-way ANOVA (here p < 0.001). ^##,^^###^Significantly different from TPG-treated and MeHg-untreated cells by a one-way Welch’s *t*-test (^##^p < 0.01, ^###^p < 0.001). ^§^Significantly different from TPG- and MeHg-untreated cells by a one-way Welch’s *t*-test (here p < 0.05). (**B**) Western blotting analysis for phospho-4EBP1 (p-4EBP1). Total cell lysates prepared at the times indicated were analyzed using the indicated antibody probes. Cropped blots are shown; all gels were run under the same experimental conditions.

The LAT family is critical for the control of protein translation and cell growth through mammalian target of rapamycin complex 1 (mTORC1) signaling, which plays a role in cap-dependent mRNA translation and is also associated with amino acid metabolism^23^. Therefore, we examined the effect of MeHg exposure and ER stress preconditioning on mTORC1 signaling. As shown in Fig. 2A, *Mtor* mRNA expression was downregulated following MeHg exposure whereas pretreatment with TPG upregulated *Mtor* expression. Consistent with this, western blot analysis demonstrated that phosphorylation of 4EBP1, a direct substrate of mTORC1, was down-regulated following exposure to MeHg and activated under ER stress preconditioning (Fig. 2B).

### The role of methionine transporters and ABCC4 on intracellular Hg content following MeHg exposure

**Figure 3:**
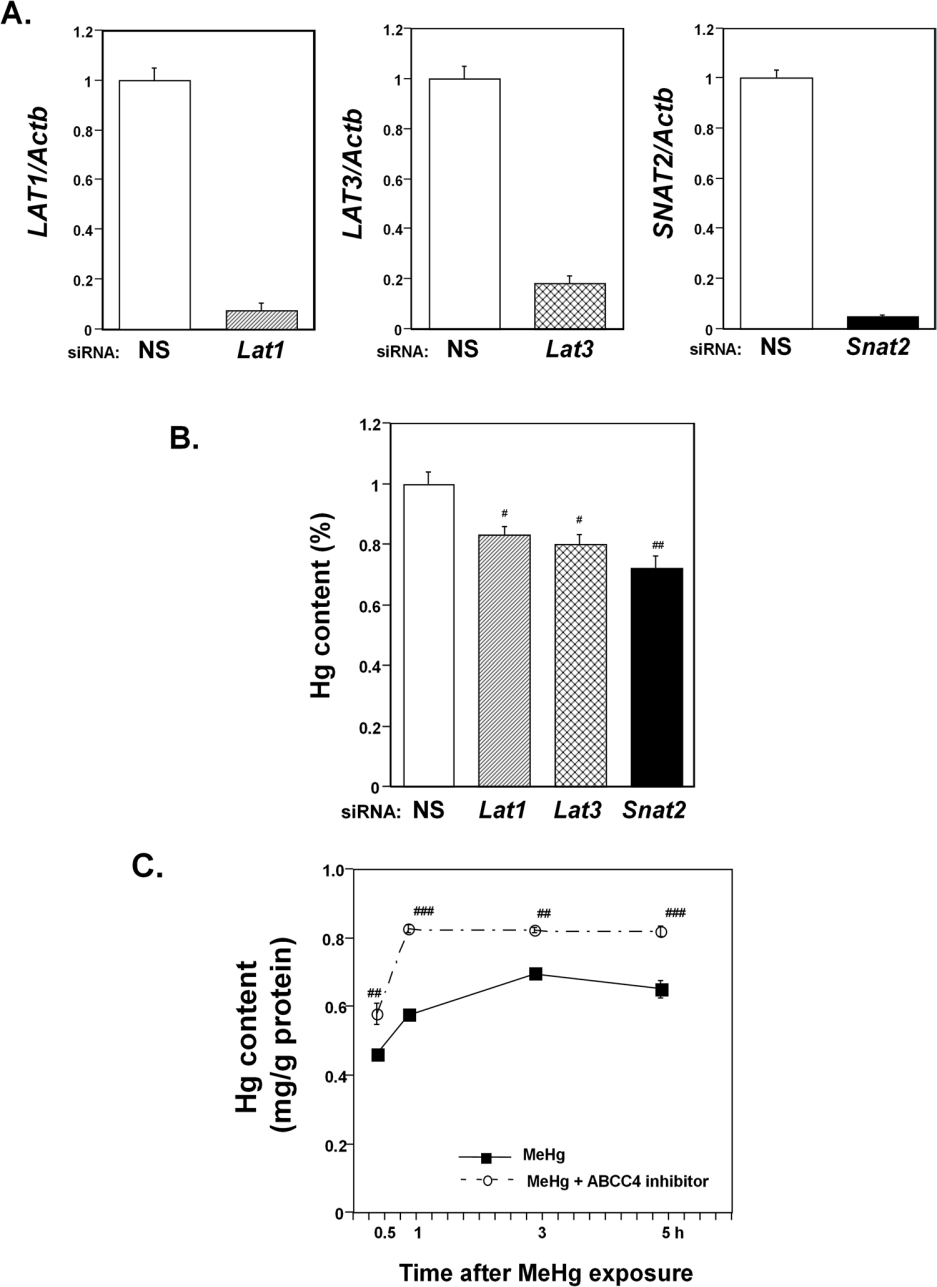
The role of membrane transporter expression for intracellular Hg content following exposure to MeHg. (**A**) Synthetic siRNA-mediated knockdown of methionine transporter *Lat1*, *Lat3*, and *Snat2*. NS, non-silencing. RT-qPCR data for each methionine transporter was shown. The histogram depicts the indicated mRNA normalized to *Actb* mRNA. Values shown are the means ± SE of 3 separate experiments. (**B**) Effect of knockdown of methionine transporters on intracellular Hg content. Cell lysates were prepared 3 h after the exposure to 0.5 μM MeHg. Averaged Hg content of NS siRNA-transfectants was regarded as 100%. Values represent the means ± SE (n = 4). ^#,^^##^Significantly different from NS siRNA-transfectants by a one-way Welch’s *t*-test (^#^p < 0.05, ^##^p < 0.01). (**C**) Effect of the ABCC4 inhibitor Ceefourin 1 on intracellular Hg content. At 5 min prior to MeHg treatment, 2 μM Ceefourin 1 was added followed by 0.5 μM MeHg. ^##,^^###^Significantly different from Ceefourin 1-untreated cells by a one-way Welch’s *t*-test (^##^p < 0.01, ^###^p < 0.001).

Next, we investigated the role of methionine transporters and ABCC4 on intracellular Hg content using synthetic siRNAs against LAT1, LAT3, or SNAT2 and the specific ABCC4 inhibitor Ceefourin 1^24^. Knockdown of *Lat1*, *Lat3*, or *Snat2* in C2C12-DMPK160 cells was confirmed by RT-qPCR (Fig. 3A). As shown in Fig. 3B, the intracellular Hg content was significantly decreased in *Lat1*, *Lat3*, and *Snat2* knockdown cells. A decrease in intracellular Hg content was corresponded to the previous data on MeHg uptake described in *Lat1* knockdown cells^15^. In contrast, the intracellular Hg content in cells treated with 2 μM Ceefourin 1 was significantly higher than that in non-treated cells (Fig. 3C). These results suggest that methionine transporters and ABCC4 are related to intracellular mercury concentration via the influx and the efflux function of MeHg, respectively, beginning at the early stages of MeHg exposure.

### Effect of Perk knockdown on membrane transporter expression

Because ER stress preconditioning induces ISR but not ATF6 and Xbp1 pathways^11^, we next analyzed the effect of each of ISR on the expression of membrane transporters in order to determine the mechanism by which prior mild ER stress upregulates these proteins.

**Figure 4:**
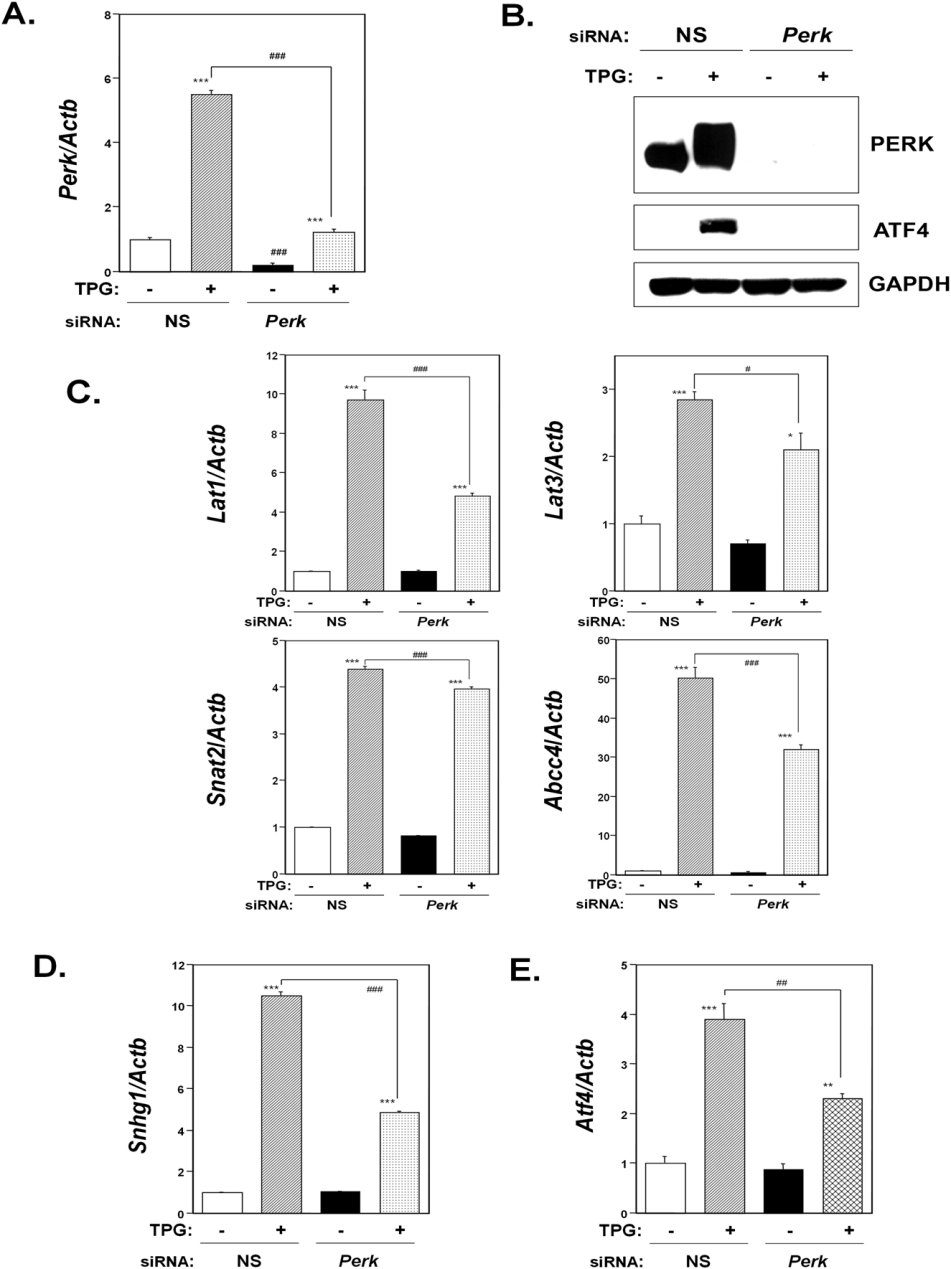
Effect of *Perk* knockdown on membrane transporter expression under ER stress preconditioning. NS, non-silencing. (**A**) RT-qPCR analysis of *Perk* mRNA. Total RNA was extracted from untreated cells or following treatment with 0.3 μg/ml TPG for 16 h. The histogram depicts *Perk* mRNA normalized to *Actb* mRNA represented as the fold increase over non-pretreated controls. Values shown are the means ± SE of 3 separate experiments. ***Significantly different from TPG-untreated cells by a one-way ANOVA (here p < 0.001). ^###^Significantly different from TPG-treated NS siRNA-transfectants by a one-way Welch’s *t*-test (here p < 0.001). (**B**) Synthetic siRNA-mediated knockdown of *Perk*. Western blots of C2C12-DMPK160 cells transfected with NS siRNA or *Perk* siRNA were analyzed using the indicated antibody probes. Cropped blots are shown; all gels were run under the same experimental conditions. (**C**–**E**) RT-qPCR analysis of membrane transporter (**C**), *Snhg1* (**D**), or *Atf4* (**E**) mRNA. NS, non-silencing. Total RNA was extracted from untreated cells or following treatment with 0.3 μg/ml TPG for 16 h. The histogram depicts the indicated mRNA normalized to *Actb* mRNA, represented as the fold increase over non-pretreated controls. Values shown are the means ± SE of 3 separate experiments. *^,^ **^,^ ***Significantly different from TPG-untreated cells by a one-way ANOVA (*p < 0.05, **p < 0.01, ***p < 0.001). ^#, ##,^^###^Significantly different from TPG-treated NS siRNA-transfectants by a one-way Welch’s *t*-test (^#^p < 0.05, ^##^p < 0.01, ^###^p < 0.001).

First, we investigated the effect of eIF2α phosphorylation on membrane transporter expression. For this, we depleted the eIF2α phosphorylation kinase PERK using siRNA knockdown methodology. Knockdown of *Perk* in C2C12-DMPK160 cells was confirmed by RT-qPCR and western blot analysis (Fig. 4A,B). As shown in Fig. 4C, pretreatment with TPG upregulated *Lat1*, *Lat3*, *Snat2*, and *Abcc4* mRNAs in both non-silencing (NS) siRNA- and *Perk* siRNA-transfected cells. However, the mRNA levels of all these transporters were significantly lower in *Perk* knockdown cells compared to NS siRNA-transfectants. The results indicated that the upregulation of these transporter mRNAs by ER stress preconditioning involved PERK expression.

Next we investigated the effect of knockdown of *Perk* on the other ISR factors, NMD suppression and ATF4 accumulation. NMD suppression was estimated for the expression of non-protein coding small nucleolar RNA host gene 1 (*Snhg1)* mRNA, which harbors a PTC. Pretreatment with TPG upregulated *Snhg1* mRNA in both NS siRNA-transfectants and *Perk* knockdown cells, suggesting that NMD was suppressed in these TPG-treated cells (Fig. 4D). However, the level of *Snhg1* mRNA was significantly downregulated in *Perk* knockdown cells compared to that in NS siRNA-transfectants. The induction of *Atf4* mRNA by ER stress preconditioning was also significantly suppressed in *Perk* knockdown cells compared to NS siRNA-transfectants (Fig. 4E).

### Effect of eIF2α phosphorylation on the membrane transporter expression

**Figure 5:**
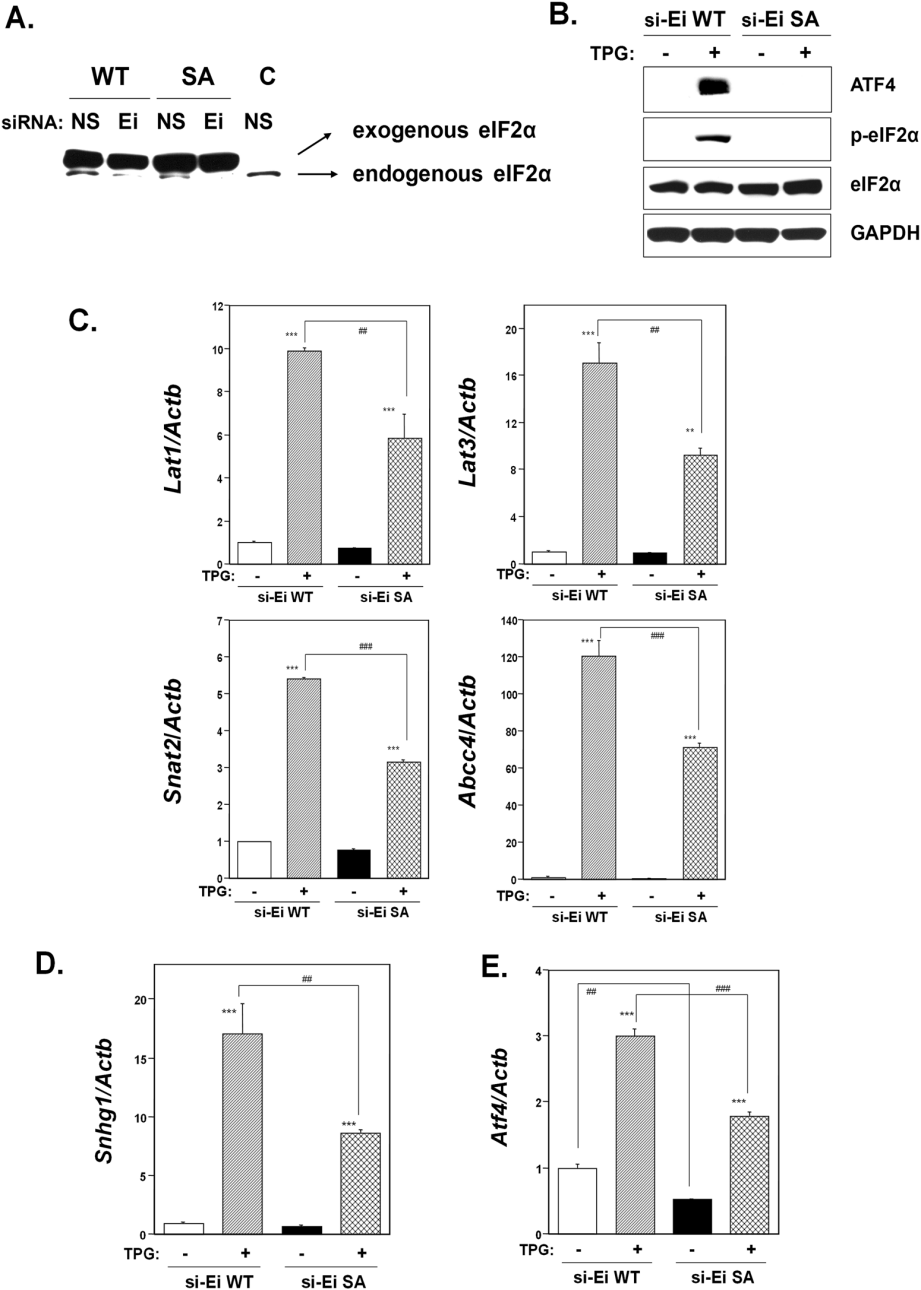
Effect of eIF2α phosphorylation on membrane transporter expression under ER stress preconditioning. WT, stable cell line transfected with wild-type eIF2α plasmid; SA, stable cell line transfected with mutant eIF2α-SA plasmid. (**A**) Synthetic siRNA-mediated knockdown of endogenous *Eif2α*. Western blots of WT and mutant SA cell lines transfected with siRNA against endogenous *Eif2α* were analyzed using anti-eIF2α antibody probes. NS, non-silencing. Ei, *Eif2α* siRNA. Representative images of 3 samples are shown. (**B**) Effect of mutant non-phosphorylatable eIF2α on ATF4, phospho-eIF2α, and eIF2α expression. Western blots of untreated endogenous *Eif2α*-knockdown WT- or SA-expressing cell lines or pretreated with 0.3 μg/ml TPG for 16 h were analyzed using the indicated antibody probes. Cropped blots shown; all gels were run under the same experimental conditions. Representative images of 3 samples are shown. (**C**–**E**) Effect of eIF2α phosphorylation on the expression of membrane transporter (**C**), *Snhg1* (**D**) or *Atf4* (**E**) mRNA was analyzed by RT-qPCR. Total RNA was extracted from untreated cells or following treatment with 0.3 μg/ml TPG for 16 h. The histogram depicts the indicated mRNA normalized to *Actb* mRNA. Values shown are the means ± SE of 4 separate experiments. **^,^ ***Significantly different from TPG-untreated cells by a one-way ANOVA (**p < 0.01, ***p < 0.001). ^##, ###^Significantly different from TPG-treated NS siRNA-transfectants by a one-way Welch’s *t*-test (^##^p < 0.01, ^###^p < 0.001).

The role of phosphorylation of eIF2α on membrane transporter expression was further investigated using mutant non-phosphorylatable eIF2α-transfected cell lines. For this, a human wild type (WT) *EIF2A* construct encoding serine at position 52 or a mutant *EIF2A*-SA construct encoding the non-phosphorylatable alanine at position 52 was transfected into C2C12-DMPK160 cells and selected by puromycin to establish stable cell lines. Endogenous eIf2α in both established cell lines was knocked down following transfection with a targeted siRNA as confirmed by western blotting (Fig. 5A). Western blot analyses confirmed the establishment of a WT cell line that expressed phospho-eIF2α under mild ER stress as well as a mutant SA cell line without phospho-eIF2α under the condition of endogenous eIf2α knockdown (Fig. 5B).

As shown in Fig. 5C, pretreatment with TPG upregulated *Lat1*, *Lat3*, *Snat2*, and *Abcc4* mRNAs in both WT and mutant SA cell lines. However, the levels of all transporters mRNAs were significantly lower in the mutant SA cell line compared to the WT cell line. The results indicated that the upregulation of these transporter mRNAs by ER stress preconditioning was dependent upon the phosphorylation of eIF2α. In addition, significant downregulation of non-protein coding *Snhg1* mRNA (Fig. 5D) and ATF4 (Fig. 5B,E) were observed in mutant SA cell line under mild ER stress as compared to the WT cell line.

### Effect of Atf4 knockdown on membrane transporter expression

**Figure 6:**
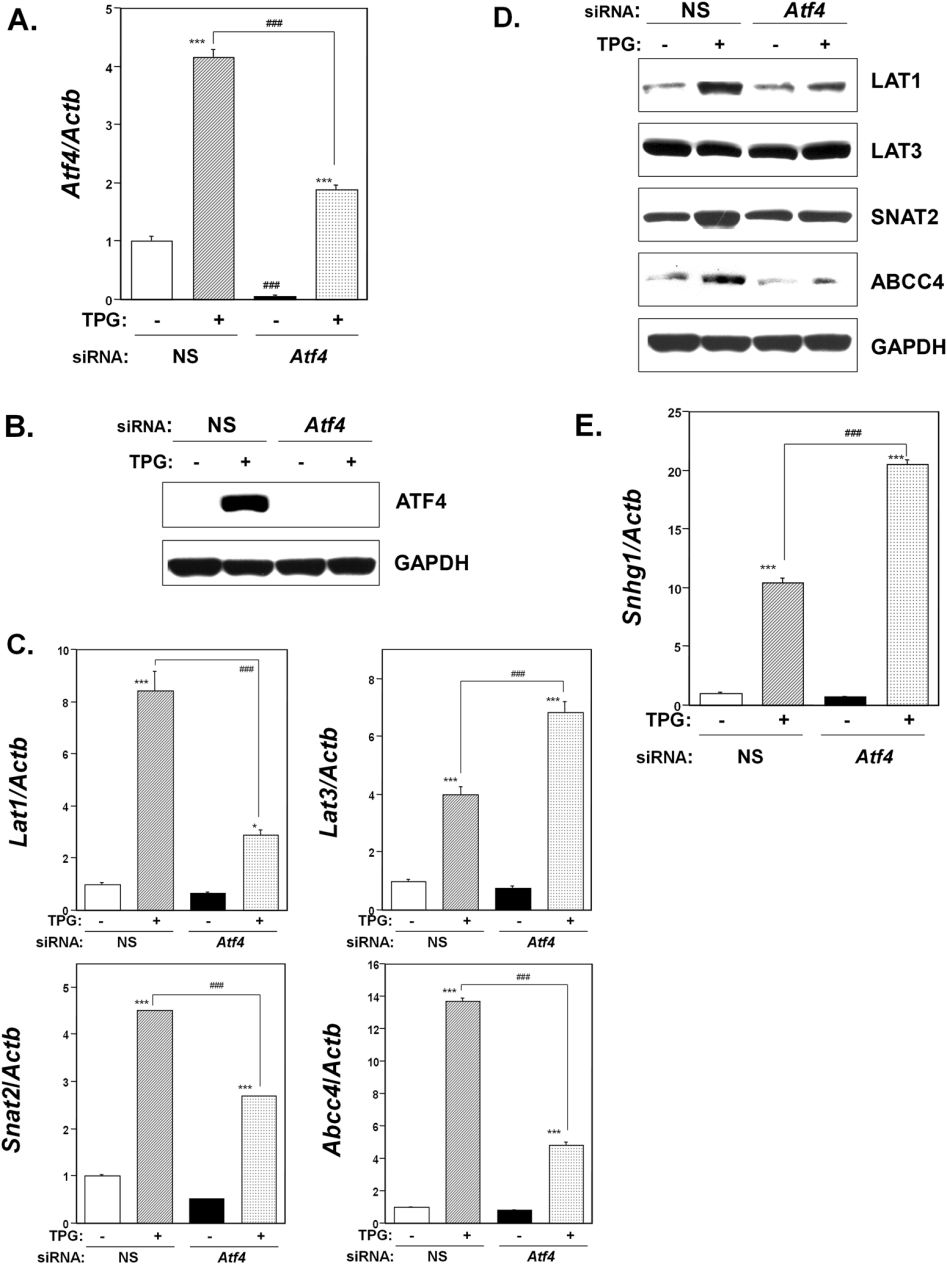
Effect of *Atf4* knockdown on membrane transporter expression under ER stress preconditioning. NS, non-silencing. (**A**) RT-qPCR analysis of *Atf4* mRNA. Total RNA was extracted from untreated cells or following treatment with 0.3 μg/ml TPG for 16 h. The histogram depicts *Atf4* mRNA normalized to *Actb* mRNA, represented as the fold increase over non-pretreated controls. Values shown are the means ± SE of 3 separate experiments. ***Significantly different from TPG-untreated cells by a one-way ANOVA (here p < 0.001). ^###^Significantly different from TPG-treated NS siRNA-transfectants by a one-way Welch’s *t*-test (here p < 0.001). (**B**) Synthetic siRNA-mediated knockdown of *Atf4*. Western blots of C2C12-DMPK160 cells transfected with NS siRNA or *Atf4* siRNA were analyzed using the indicated antibody probes. Cropped blots are shown; all gels were run under the same experimental conditions. (**C**,**E**) Effect of *Atf4* knockdown on the expression of membrane transporter (**C**) or *Snhg1* (**E**) mRNA was analyzed by RT-qPCR. Total RNA was extracted from untreated cells or following treatment with 0.3 μg/ml TPG for 16 h. The histogram depicts the indicated mRNA normalized to *Actb* mRNA. Values shown are the means ± SE of 3 separate experiments. *^,^ ***Significantly different from TPG-untreated cells by a one-way ANOVA (*p < 0.05, ***p < 0.001). ^###^Significantly different from TPG-treated NS siRNA-transfectants by a one-way Welch’s *t*-test (here p < 0.001). (**D**) Western blots of C2C12-DMPK160 cells transfected with NS siRNA or *Atf4* siRNA were analyzed using the indicated antibody probes. Cropped blots are shown; all gels were run under the same experimental conditions.

Next, we investigated the effect of knockdown of *Atf4*, a transcriptional activator that modulates a wide spectrum of downstream genes involved in adaptation to stresses, on LAT1, LAT3, SNAT2, and ABCC4 expression in preconditioned cells. Knockdown of *Atf4* in C2C12-DMPK160 cells was confirmed by RT-qPCR and western blotting (Fig. 6A,B). As shown in Fig. 6C, pretreatment with TPG upregulated *Lat1*, *Lat3*, *Snat2*, and *Abcc4* mRNAs in both NS siRNA- and *Atf4* siRNA-transfected cells. However, the levels of *Lat*1, *Snat2*, and *Abcc4* mRNAs were significantly downregulated in *Atf4* knockdown cells compared to those in NS siRNA-transfectants, whereas the level of *Lat3* mRNA was significantly upregulated in *Atf4* knockdown preconditioned cells compared to NS siRNA-transfected preconditioned cells. The results of western blot analysis showed a decrease in the expression of LAT1, SNAT2, and ABCC4 proteins and an increase in LAT3 in *Atf4* knockdown preconditioned cells compared to NS siRNA-transfected preconditioned cells (Fig. 6D). The results suggested that the upregulation of LAT1, SNAT2, and ABCC4 caused by ER stress preconditioning involved ATF4 whereas LAT3 upregulation did not. Pretreatment with TPG upregulated *Snhg1* mRNA in both NS siRNA-transfectants and *Atf4* knockdown cells. However, the level of *Snhg1* mRNA was significantly upregulated in *Atf4* knockdown cells compared to NS siRNA-transfectants (Fig. 6E), suggesting that ER stress preconditioning-mediated NMD suppression was not a downstream event of Atf4 accumulation.

### Effect of NMD suppression on membrane transporter expression

**Figure 7:**
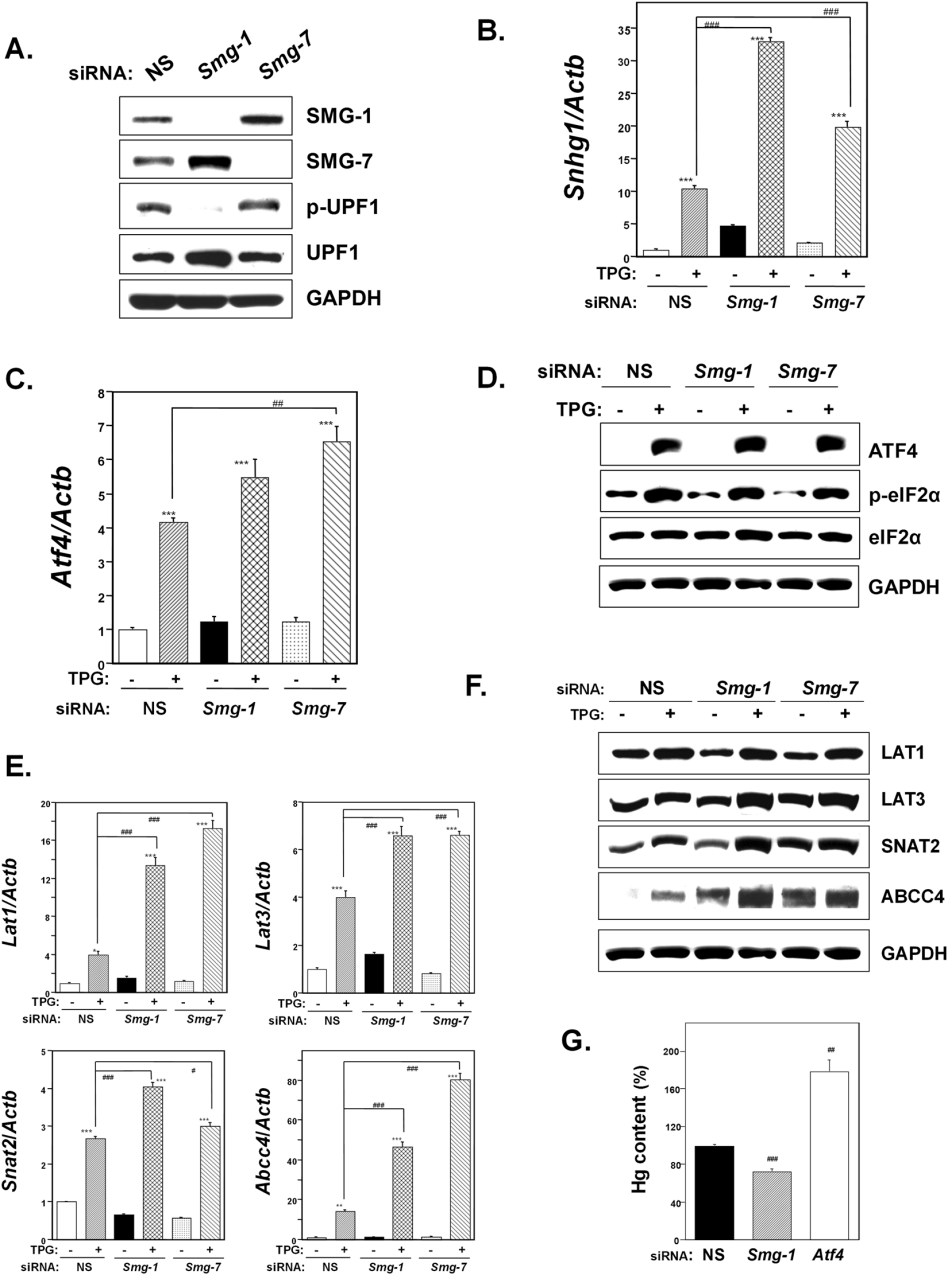
Effect of knockdown of the NMD component *Smg-1* or *Smg-7* on membrane transporter expression. NS, non-silencing. (**A**) Western blot analysis for NMD components. *Smg-1* or *Smg-7* siRNA−transfected cells were analyzed using the indicated antibody probes. Cropped blots are shown; all gels were run under the same experimental conditions. (**B**,**C**) RT-qPCR analysis of *Snhg1* (**B**) or *Atf4* (**C**) mRNA. Total RNA was extracted from untreated cells or following treatment with 0.3 μg/ml TPG for 16 h. The histogram depicts each mRNA normalized to *Actb* mRNA, represented as the fold increase over non-pretreated NS transfectants. Values shown are the means ± SE of 3 separate experiments. ***Significantly different from TPG-untreated cells by a one-way ANOVA (here p < 0.001). ^###^Significantly different from TPG-treated NS siRNA-transfectants by a one-way Welch’s *t*-test (here p < 0.001). (**D**) Western blots of C2C12-DMPK160 cells transfected with NS, *Smg-1*, or *Smg-7* siRNA were analyzed using the indicated antibody probes. Cropped blots are shown; all gels were run under the same experimental conditions. (**E**) Effect of *Smg-1* or *Smg-7* knockdown on membrane transporter mRNA expression analyzed by RT-qPCR. Total RNA was extracted from untreated cells or treatment with 0.3 μg/ml TPG for 16 h. The histogram depicts the indicated mRNA normalized to *Actb* mRNA. Values shown are the means ± SE of 4 separate experiments. *^,^ **^,^ ***Significantly different from TPG-untreated cells by a one-way ANOVA (*p < 0.05, **p < 0.01, ***p < 0.001). ^#, ###^Significantly different from TPG-treated NS siRNA-transfectants by a one-way Welch’s *t*-test (^#^p < 0.05, ^###^p < 0.001). (**F**) Western blots for membrane transporters of C2C12-DMPK160 cells transfected with NS, *Smg-1*, or *Smg-7* siRNA were analyzed using the indicated antibody probes. Cropped blots are shown; all gels were run under the same experimental conditions. (**G**) Effect of *Smg-1* or *Atf4* knockdown on intracellular Hg content. Cells transfected with NS, *Smg-1*, or *Atf4* siRNA were pretreated with TPG (0.2 μg/ml) for 16 h and then exposed to 0.5 μM MeHg. Cell lysates were prepared 1 h after the exposure to MeHg. Averaged Hg content of NS siRNA-transfectants was regarded as 100%. Values represent the means ± SE (n = 4). ^##, ###^Significantly different from TPG-treated NS siRNA-transfectants by a one-way Welch’s *t*-test (^##^p < 0.01, ^###^p < 0.001).

To investigate the effect of NMD suppression, another ISR factor, on the expression of membrane transporters, knockdown of the NMD components *Smg-1* or *Smg-7* was performed. Knockdown of *Smg-1* or *Smg-7* in each siRNA-transfected C2C12-DMPK160 cell type was confirmed by western blotting (Fig. 7A). NMD inhibition was supported by the decrease (following *Smg-1* knockdown) or increase (following *Smg-7* knockdown) in phospho (p)-UPF1, a central component of NMD, for which the UPF1 phosphorylation and dephosphorylation cycle is essential^25^. In both *Smg-1* and *Smg-7* knockdown cells, significant upregulation of *Snhg1* mRNA was observed compared to NS siRNA-transfectants and the level was further upregulated by ER stress preconditioning (Fig. 7B). In addition, the induction of *Atf4* mRNA by ER stress preconditioning was higher in NMD-suppressed cells compared to NS siRNA-transfected preconditioned cells and significantly higher in *Smg-7* knockdown cells (Fig. 7C). Western blot analysis confirmed that phosphorylation of eIF2α and ATF4 accumulation in preconditioned NS- or NMD suppressed-cells (Fig. 7D).

As shown in Fig. 7E, pretreatment with TPG upregulated *Lat1*, *Lat3*, *Snat2*, and *Abcc4* mRNAs in both NS siRNA-transfectants and NMD-suppressed cells. However, the levels of these transporter mRNAs were significantly higher in NMD suppressed cells compared to NS siRNA-transfectants. Western blot analyses showed an increase in the expression of LAT3, SNAT2, and ABCC4 in NMD-suppressed preconditioned cells compared to NS siRNA-transfected preconditioned cells (Fig. 7F). The results suggested that the upregulation of these membrane transporters by mild ER stress involved NMD suppression. A decrease in intracellular Hg content was confirmed in *Smg-1* knockdown preconditioned cells whereas that was increased in *Atf4* knockdown cells (Fig. 7G).

## Discussion

In this study, we demonstrated that ER stress preconditioning increased both the cellular influx and the efflux of MeHg and modified intracellular Hg content. RT-qPCR and western blot analyses of the expression of membrane transporters that affect cellular influx (LAT1, LAT3, SNAT2), and efflux (ABCC4) of MeHg, revealed that all of these membrane transporters mRNAs and LAT1, SNAT2, and ABCC4 proteins were upregulated and that the upregulation of ABCC4 was exceptionally high (Fig. 1C,E). The results of siRNA study for methionine transporters and ABCC4 inhibition study suggested that methionine transporters and ABCC4 represented a critical factor for the influx and efflux of MeHg, respectively (Fig. 3). Together with the time course study of intracellular Hg content (Fig. 1A) and membrane transporter expression after the exposure to MeHg (Fig. 1B), the findings indicated that the efflux of MeHg was activated than the influx after the exposure to MeHg and that this phenomenon was further enhanced by ER stress preconditioning. The results thus suggested that ER stress preconditioning alleviated the load of MeHg on cells through the enhanced efflux of MeHg caused by highly upregulated expression of ABCC4.

It has been reported that the induction of LAT family proteins and SNAT2 is coincident with mTORC1 pathway activation. However, our study demonstrated that MeHg downregulated the mTORC1 pathway despite the upregulation of LAT1 and SNAT2 expression (Figs 1B,E and 2A,B). As intracellular amino acid availability is recognized as a major mTORC1 regulatory mechanism^26^, the MeHg-induced inactivation of mTORC1 signaling suggested the likelihood of poor amino acid metabolism in MeHg-exposed cells. Notably, MeHg is easily incorporated in sulfur-containing amino acid cysteine and can thereby be transported to cells as a MeHg-cysteine complex, which might disturb amino acid availability. In turn, a defect of amino acid availability might underlie in part the loss of body weight exhibited by MeHg-exposed rats^8,27^.

We also identified that ER stress preconditioning protected against MeHg-induced mTORC1 signaling inactivation (Fig. 2A,B). Because the protective effect of ER stress preconditioning against MeHg cytotoxicity is due to the induction of ISR^11^, we investigated the role of ISR including eIF2α phosphorylation, accumulation of ATF4, and NMD suppression in the upregulation of membrane transporters in order to determine the mechanism by which prior ER stress upregulates membrane transporter expression. The results of siRNA-mediated knockdown study of *Perk*, *Atf4*, or NMD components and the study using mutant non-phosphorylatable eIF2α-transfected cells uncovered that NMD suppression was related to the upregulation of LAT3 expression and that ATF4 accumulation led to upregulated LAT1, SNAT2, and ABCC4 expression (Figs 4–7). Our results are consistent with a prior report that ATF4 regulates the expression of the amino acid transporters LAT1 and SNAT2^28–30^.

**Figure 8:**
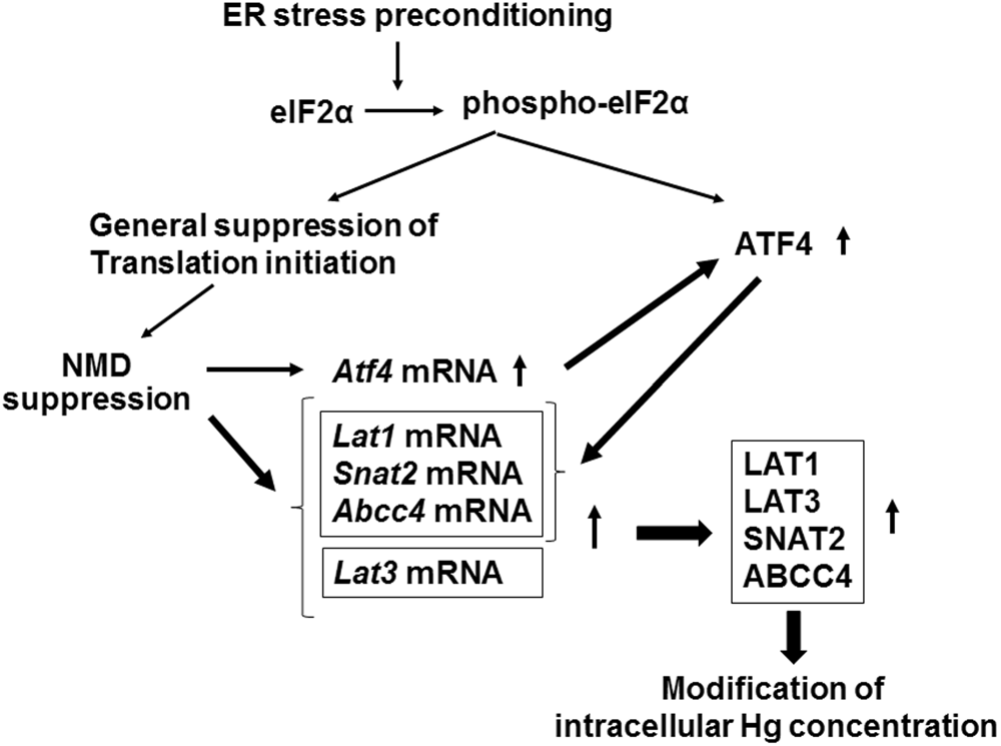
Summarized membrane transporter upregulation induced by ER stress preconditioning. NMD suppression was related to the upregulation of LAT3 expression and ATF4 accumulation led to upregulated LAT1, SNAT2, and ABCC4 expression. ER stress preconditioning amplified membrane transporter expression most likely through the translation of the upregulated mRNAs caused by ATF4-dependent transcription and NMD suppression.

We further discovered that ATF4 accumulation upregulated ABCC4. The functional significance of ABCC4 upregulation was supported by an increase in intracellular Hg content in *Atf4* knockdown preconditioned cells (Fig. 7G). In addition, we showed NMD suppression upregulated LAT3. In turn, ER stress preconditioning amplified membrane transporter expression most likely through the translation of the upregulated membrane transporter mRNAs caused by ATF4-dependent transcription and NMD suppression. We confirmed a decrease in intracellular Hg content in *Smg-1* knockdown preconditioned cells (Fig. 7G). Our findings were summarized in Fig. 8. Notably, the identification of these mRNAs as targets of NMD is consistent with its role in regulating transcripts encoding proteins involved in amino acid metabolism and transport that carry various traits predisposing them to errors in translation^19^. In particular, among the transporters examined in this study, it has been reported that *LAT1* contains uORFs and exon with PTC^19^ and that alternatively spliced transcripts of *Abcc4* (*Abcc4s*) bear nonsense codons^31^. Accordingly, we also found that ER stress preconditioning increased alternatively spliced transcripts of *Abcc4* (Fig. 1D). Additionally, it is expected that *Lat3* also contains uORF, 3′UTR exon and/or uncharacterized feature of NMD substrate^32^ because *Lat3* mRNA and LAT3 could only be upregulated through NMD suppression (Figs 6C,D and 7E,F).

Notably, the results of a microarray study comparing control cells to those with knockdown of the NMD component *Upf1* suggested that almost of 10% of non-mutated mRNAs are regulated by NMD^19^. The diverse transcripts induced by NMD suppression included mRNAs that play an important part in mediating the unfolded protein response, integrated stress response, and in amino acid transport and metabolism, as well as proto-oncogene mRNAs. In the current study, we also demonstrated that NMD suppression under ER stress preconditioning upregulated membrane transporter mRNAs related to the influx and efflux of MeHg as well. Furthermore, NMD suppression under ER stress preconditioning upregulated ATF4 mRNA and amplified the accumulation of ATF4 through eIF2α phosphorylation-mediated translation facilitation of ATF4 protein-coding ORF, resulting in the transcription facilitation of the membrane transporter mRNAs (Fig. 8).

Finally, it has been reported that one-third of alternative transcripts examined contain a PTC that renders them targets for NMD^33^. It is considered therefore that NMD suppression induced by a variety of stresses should stabilize many of these NMD-candidate transcripts. The involvement of these NMD-candidate transcripts in proteome diversity consequent to varied stresses remains to be elucidated; however, the findings of the current study support the potential of NMD regulation as a tool to dynamically alter gene expression and protect cells against environmental stresses.

In conclusion, we have demonstrated that ER stress preconditioning resulted in the upregulation of membrane transporters through the activation of the phospho-eIF2α/ ATF4 pathway and NMD suppression, leading to a decrease in intracellular Hg content. ER stress preconditioning also improved intracellular amino acid metabolism. Together, the results indicate that intracellular Hg content is able to be modulated through membrane transporter upregulation consequent to promoting the activation of the phospho-eIF2α/ATF4 pathway and NMD suppression. Therefore, these factors may represent therapeutic targets for the alleviation of MeHg cytotoxicity by inducing not only protective stress responses but also enhanced efflux of elevated levels of Hg.

## Methods

### Cell culture and drug treatments

The mouse myogenic C2C12-DMPK160 cell line, which is susceptible to MeHg treatment^34^, was cultured in Dulbecco’s modified Eagle’s medium (Nissui Pharmaceuticals) supplemented with 10% fetal bovine serum (HyClone), 300 μg/ml glutamine (Nissui Pharmaceuticals) and 0.4 mg/ml Geneticin (G418) (Thermo Fisher Scientific) and exposed to MeHg in serum-free Cosmedium (Cosmo Bio Co., Ltd) as described previously^35^. Thapsigargin (TPG) (Sigma-Aldrich) was prepared as described previously^11^. Ceefourin 1 (Abcam) stock was dissolved in dimethylsulfoxide (WAKO). Ceefourin 1 was added 5 min prior to MeHg treatment. For the preconditioning study, 0.3 µg/ml TPG was added to the cells for 16 h prior to MeHg treatment. After removal of TPG, cells were exposed to MeHg as described previously^35^.

### Measurement of intracellular mercury concentration

Preconditioned or non-preconditioned cells (1.5 × 10^5^ cells/35-mm dish) were exposed to MeHg. At the indicated time of MeHg exposure, cells were washed twice with Ca^2+^- and Mg^2+^-free phosphate buffered saline (PBS (−)) and incubated on ice for 10 min in Cell Lysis buffer (Cell Signaling Technologies) containing complete protease inhibitor cocktail (Roche). Collected cell lysates were homogenized using a QIA shredder (Qiagen). Total Hg concentrations of cell lysates were determined by the oxygen combustion-gold amalgamation method as described previously^10^. A duplicate assay was performed per each three dishes at the indicated time. The protein content was measured using a DC protein assay kit (Bio-Rad Laboratories).

### RT-qPCR

Total RNA was extracted and first-strand cDNA was prepared as described previously^34^. qPCR was performed using a LightCycler DX 400 System (Roche). *Lat1, Lat3, Snat2, Abcc4, Abcc4s, Snhg1*, *Atf4*, *Perk*, and *Mtor* mRNAs were amplified using a SYBR Green Master Mix (Roche) and specific primer sets. Specific primer sets for *Snhg1* and *Atf4* have been described previously^11^. Additional primer sets used were as follows: *LAT1* (Gene Bank accession number AB023409) 5′-TCTTCGCCACCTACTTGCTC-3′ (nucleotides 463−482) and 5′-CGCATCACCTTGTCCCATGT-3′ (nucleotides 695−676), *LAT3* (AB103034) 5′-CAGGAAAAGATGCTCAACCT-3′ (nucleotides 470−489) and 5′-CCATGAAAGTGGATCGCAAA-3′ (nucleotides 761−742), *Snat2* (NM_175121) 5′-CTGGAAGAC- GTCTGGTAGTG-3′ (nucleotides 3583−3602) and 5′-CAGGATCCTGGTTGTCATGG-3′ (nucleotides 3850−3831), *Abcc4* (BC150822) 5′-TGGACTTCATCCAGACGTTG-3′ (nucleotides 2586−2605) and 5′-TCTCCTCAGCTTTGTAAGCC-3′ (nucleotides 2828−2809), *Abcc4s* (AF530634 + AF530635) 5′-CCTCCTGTGTTCAAGCAGTC-3′ (nucleotides 67−86) and 5′-TCTTCCAGTCTCCGCTTATG-3′ (nucleotides 314−295), *Mtor* (NM_020009) 5′-CCAGGA- TACACTAAGAGTCC-3′ (nucleotides 5820−5839) and 5′-CGACCAATATCTGTGAGAAG-3′ (nucleotides 6017−5998), and *Perk* (NM_010121) 5′-CGAAGCTTCTCCCTATACCC-3’ (nucleotides 2560−2579) and 5′-TAGAGTGGTTGGCCTGGTAG-3′ (nucleotides 2782−2763). Transcript levels were normalized to *Actb* mRNA as described previously^34^.

### Western blot analysis

Western blotting was performed as previously described^36^. Briefly, cells were incubated on ice for 10 min in Cell Lysis buffer containing complete protease inhibitor cocktail. Next, the cells were harvested and homogenized using a QIA shredder. Then, samples were separated by 10% sodium dodecyl sulfate-polyacrylamide gel electrophoresis (SDS-PAGE) for analysis of LAT1, LAT3, SNAT2, eIF2α, phospho-eIF2α, phospho-4EBP1, 4EBP1, ATF4, and glyceraldehyde 3-phosphate dehydrogenase (GAPDH) in the presence of dithiothreitol (Sigma-Aldrich). For the components of NMD (phospho-UPF1, UPF1, SMG-1, and SMG-7), PERK, and ABCC4, 5% SDS-PAGE was adopted. The gels were transferred to nitrocellulose membranes (Bio-Rad). The membrane was blocked in EzBlock Chemi (ATTO) for 30 min, and then incubated with the indicated antibody probes purchased from the following suppliers: anti-eIF2α, anti-phospho-eIF2α, anti-ATF4, anti-phospho-4EBP1, anti-4EBP1, anti-PERK, and anti-GAPDH (Cell Signaling Technologies); anti-LAT1 (Sigma); anti-ABCC4 and anti-SNAT2 (Abcam); anti-LAT3 (Nobus Biologicals). The antibodies of NMD components SMG-1, SMG-7, UPF1, and pUPF1 have been described elsewhere^25,37–39^. The proteins were detected by a chemiluminescence system with Clarity Western ECL Substrates (Bio-Rad Laboratories) or EzWestLumi plus (ATTO).

### siRNA, plasmid preparation, and transfection

Mouse *Smg-1* and *Smg-7* siRNAs have been described previously^34^. Mouse *Eif2α* (target sequence: 5′-CAAUGUUGUUAUGUUCU-3′), *Lat1* (target sequence: 5′-AGUGAAAGAGCAAGCCCAA-3′), *Lat3* (target sequence: 5′-UGAAAAAGACCAAACUCAU-3′), and *Snat2* (target sequence 5′-UGUUAGCGUCGGCAUUCAA-3′) siRNAs were designed using i-Score Designer^40^ and asymmetric siRNA^41^ were synthesized (GeneDesign, Inc.). Other synthetic siRNAs were purchased from Qiagen: mouse *Abcc4*, FlexiTube siRNA SI02833019; mouse *Atf4*, FlexiTube siRNA SI00905905; mouse *Perk*, FlexiTube siRNA SI01319269; and the All Star Negative Control siRNA. Synthetic siRNA transfections were carried out using Lipofectamine RNAiMAX (Thermo Fisher Scientific) and the cells were analyzed 48−64 h after transfection. The results were confirmed in more than 3 independent experiments.

The plasmid-expressed human eIF2α-S52A mutant was constructed by site-directed mutagenesis of pSR-strep-HA-eIF2α^38^. Subsequently, a puromycin resistance gene expression cassette derived from silentGene-puro (Promega) was inserted into wild-type (WT) and S52A mutant eIF2α expression (SA) plasmids to generate pSR-strep-HA- eIF2α_puro and pSR-strep-HA-eIF2α-S52A_puro. The transfections of plasmid siRNAs were carried out using Lipofectamine 3000 (Thermo Fisher Scientific) and stable cell lines were selected by puromycin (Sigma-Aldrich).

### Statistical analysis

Statistical analysis was conducted by using Graph Pad PRISM 5.0 (GraphPad Software). Data were analyzed by a one-way ANOVA for multiple data analyses or a one-way Welch’s *t*-test for comparison between two data sets and are expressed as the means ± SEM. A difference was considered statistically significant when p < 0.05.

## Electronic supplementary material

Supplementary Information
