# Supplementary material for: Endoplasmic reticulum stress preconditioning modifies intracellular mercury content by upregulating membrane transporters
Source: Sci Rep. 2017 Sep 28;7:12390. doi: 10.1038/s41598-017-09435-3 (PMC5620048; doi:10.1038/s41598-017-09435-3)

Endoplasmic reticulum stress preconditioning modifies intracellular mercury content by upregulating membrane transporters

Fusako Usuki[1,*], Masatake Fujimura[2], Akio Yamashita[3]

[1]Department of Clinical Medicine and [2]Basic Medical Sciences, National Institute for Minamata Disease, Kumamoto 867-0008, Japan

[3]Department of Molecular Biology, Yokohama City University School of Medicine, Yokohama 236-0004, Japan

[*]To whom correspondence should be addressed. Tel: +81 966 63 3111; Fax: +81 966 61 1145; E-mail: usuki@nimd.go.jp

Supplemental Fig. 1E

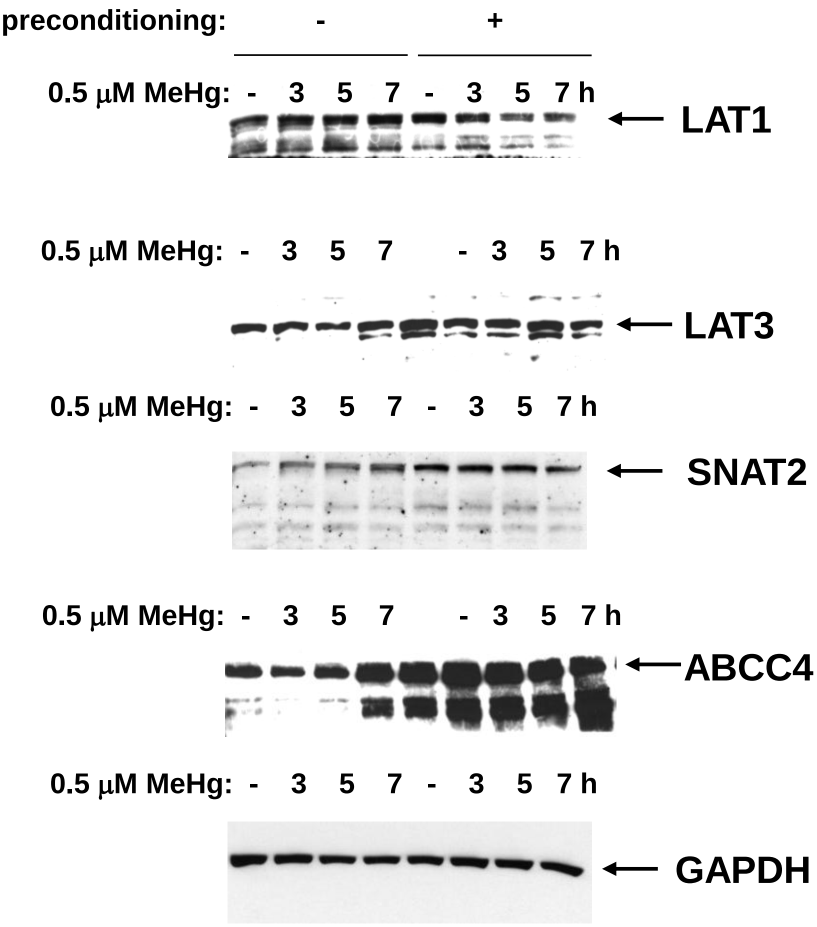

Supplemntal Fig. 2B

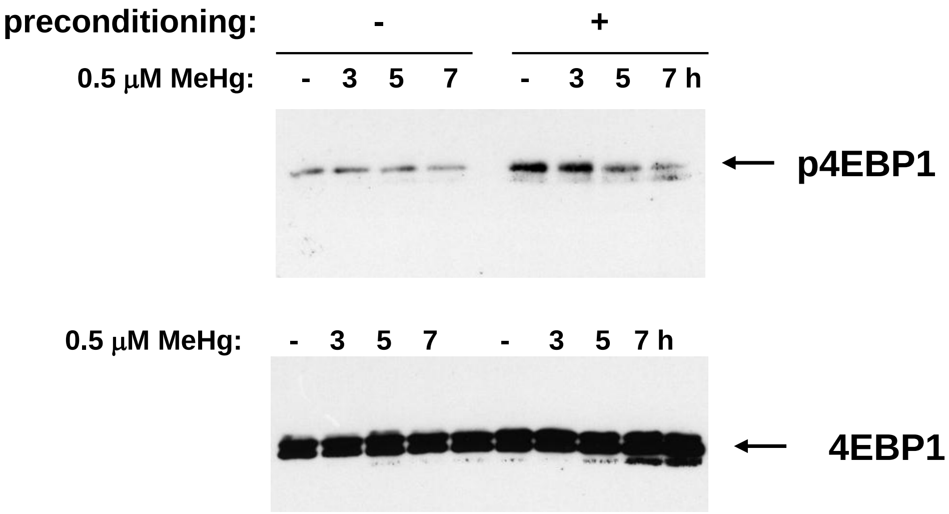

Supplemental Fig. 4B

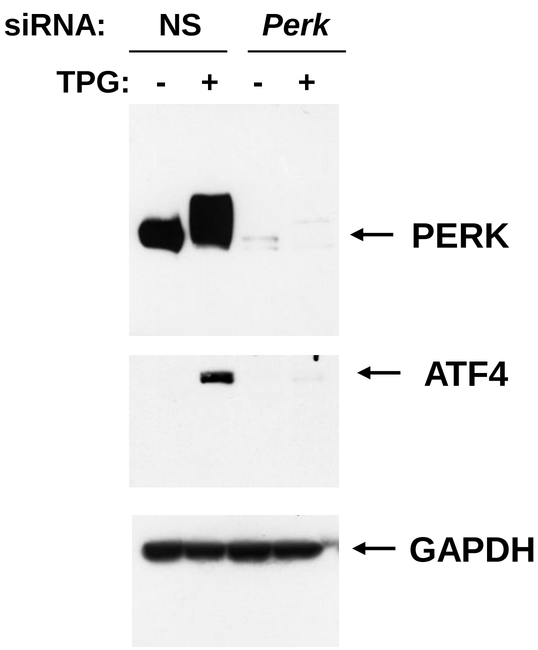

Supplemental Fig. 5A

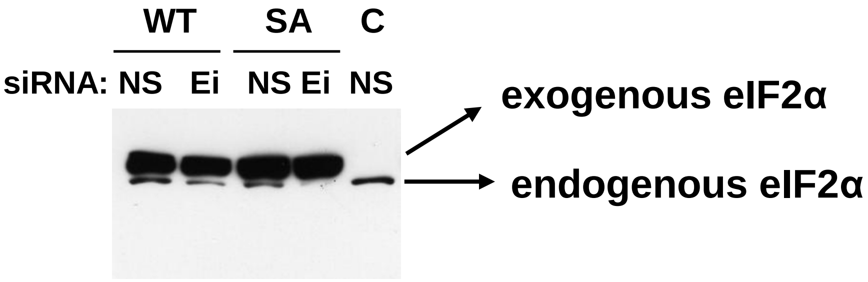

Supplemental Fig. 5B

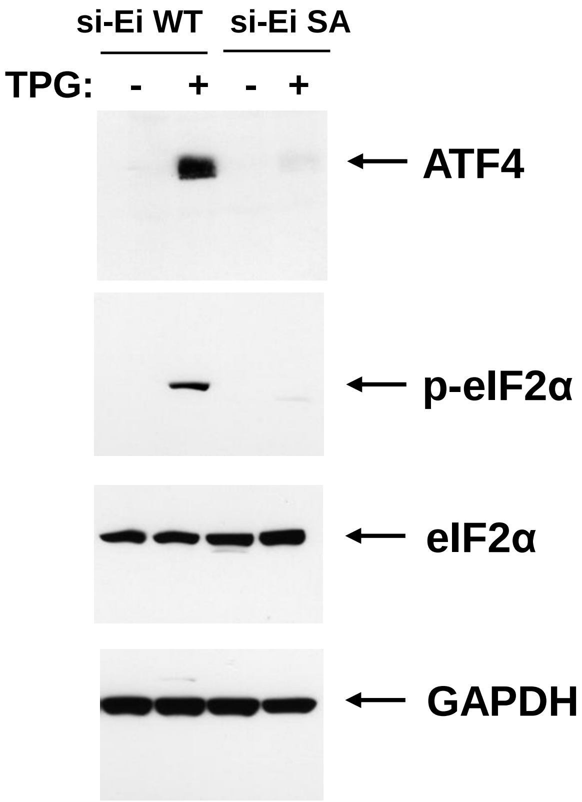

Supplemental Fig. 6B

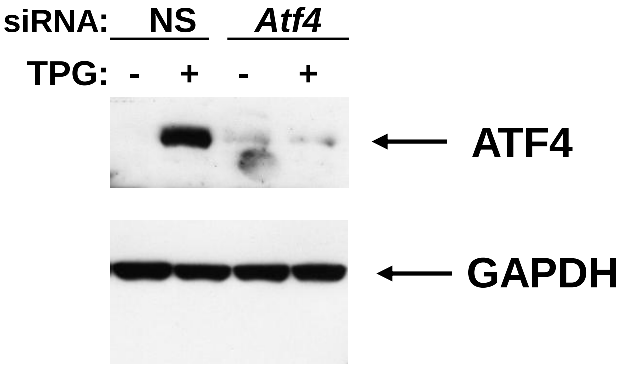

Supplemental Fig. 6D

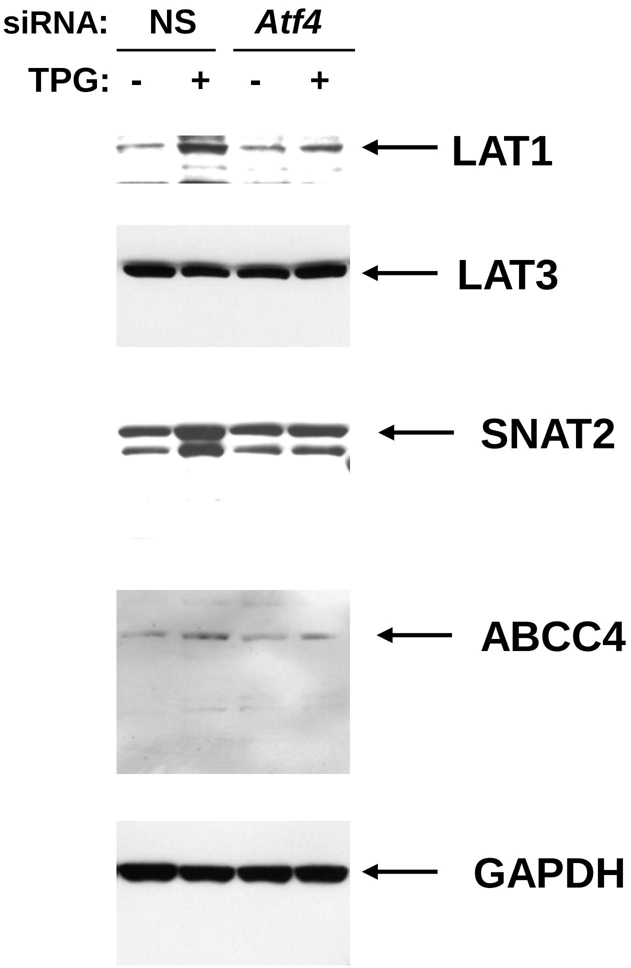

Supplemental Fig. 7A

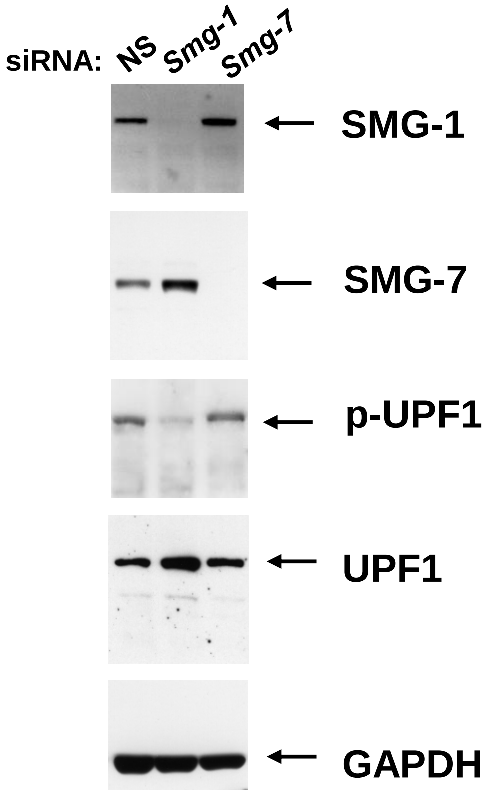

Supplemental Fig. 7D

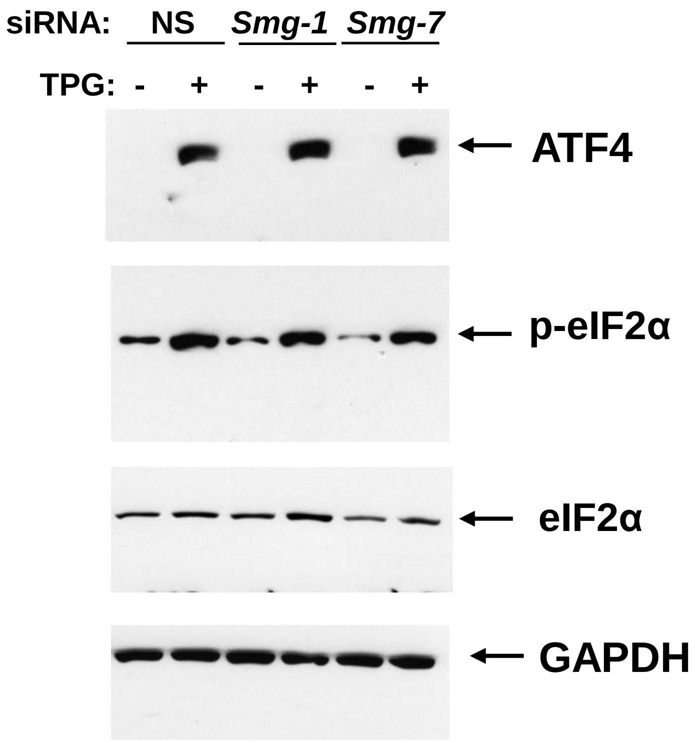

Supplemental Fig. 7F

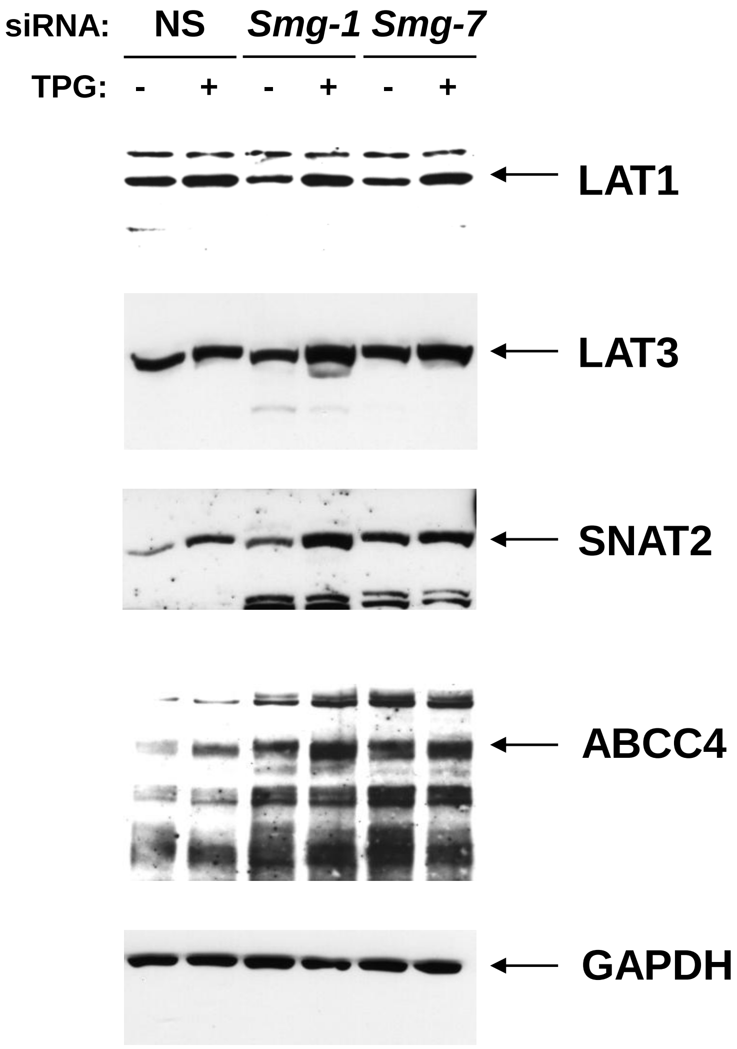

Supplement: Supplementary file 1 — Supplementary Information [file 41598_2017_9435_MOESM1_ESM.pdf]
